# Engineered domain-inlaid Nme2Cas9 adenine base editors with increased on-target DNA editing and targeting scope

**DOI:** 10.1186/s12915-023-01754-4

**Published:** 2023-11-09

**Authors:** Ding Zhao, Xun Gao, Jiale Zhou, Jinze Li, Yuqiang Qian, Di Wang, Wenchao Niu, Tao Zhang, Mingyang Hu, Haoyang Xiong, Liangxue Lai, Zhanjun Li

**Affiliations:** 1 Jilin Provincial Key Laboratory of Animal Embryo Engineering, College of Veterinary Medicine, Jilin University, Changchun, 130062 China; 2 CAS Key Laboratory of Regenerative Biology, Guangdong Provincial Key Laboratory of Stem Cell and Regenerative Medicine, South China Institute for Stem Cell Biology and Regenerative Medicine, Guangzhou Institutes of Biomedicine and Health, Chinese Academy of Sciences, Guangzhou, 510530 China; 3 Guangzhou Regenerative Medicine and Health Guang Dong Laboratory (GRMH-GDL), Guangzhou, 510005 China; 4 Institute for Stem Cell and Regeneration, Chinese Academy of Sciences, Beijing, 100101 China

**Keywords:** Adenine base editors, Inlaid domain, Nme2ABE8e-797, Nme2ABE8e-797^Smu^, Nme2ABE8e-797^−C^

## Abstract

**Background:**

Nme2ABE8e has been constructed and characterized as a compact, accurate adenine base editor with a less restrictive dinucleotide protospacer-adjacent motif (PAM: N4CC) but low editing efficiency at challenging loci in human cells. Here, we engineered a subset of domain-inlaid Nme2Cas9 base editors to bring the deaminase domain closer to the nontarget strand to improve editing efficiency.

**Results:**

Our results demonstrated that Nme2ABE8e-797 with adenine deaminase inserted between amino acids 797 and 798 has a significantly increased editing efficiency with a wide editing window ranging from 4 to 18 bases in mammalian cells, especially at the sites that were difficult to edit by Nme2ABE8e. In addition, by swapping the PAM-interacting domain of Nme2ABE8e-797 with that of SmuCas9 or introducing point mutations of eNme2-C in Nme2ABE8e-797, we created Nme2ABE8e-797^Smu^ and Nme2ABE8e-797^−C^, respectively, which exhibited robust activities at a wide range of sites with N4CN PAMs in human cells. Moreover, the modified domain-inlaid Nme2ABE8e can efficiently restore or install disease-related loci in Neuro-2a cells and mice.

**Conclusions:**

These novel Nme2ABE8es with increased on-target DNA editing and expanded PAM compatibility will expand the base editing toolset for efficient gene modification and therapeutic applications.

**Supplementary Information:**

The online version contains supplementary material available at 10.1186/s12915-023-01754-4.

## Introduction

Adenine base editors (ABEs), which contain an evolved adenosine deaminase and a Cas9 nickase (nCas9), enable efficient conversion of A-to-G nucleotides without accompanying double-stranded DNA break (DSB) generation [14]. Upon targeting recognition via single-guide RNA (sgRNA), ABEs deaminate specific bases within a defined editing window relative to the PAM in the nontarget strand. Nme2Cas9, a Cas9 ortholog from *Neisseria meningitidis*, has been developed as a compact gene editing tool with high precision [12, 20]. Nme2CBEs have been proven to be reliable for base editing, but no significant base editing events were observed using Nme2ABEmax in a previous study [26]. Recently, two groups have been involved in the development and characterization of Nme2ABE8e in mammalian cells [8, 38], with single-AAV delivery achieved in a mouse disease model [38]. However, Nme2ABE8e has a low editing efficiency at challenging loci in human cells. Considering the potential limitation of adenosine deaminase access to adenine on the displaced nontarget strand as SpCas9 [3], a series of domain-inlaid Nme2Cas9 base editors were designed to further improve the editing activity of Nme2-ABE8e.

It has been demonstrated that swapping the PAM-interacting domain between closely related Cas9 orthologs can result in a chimera that recognizes distinct PAMs [6, 12, 16, 37]. Additionally, a Nme2Cas9 variant named eNme2-C, which was developed although phage-assisted continuous evolution, enables efficient genome editing at PAMs with a single specific pyrimidine nucleotide [18]. To engineer the improved Nme2ABE8e-797^Smu^ and Nme2ABE8e-797^−C^, we swapped the PAM-interacting domain of Nme2ABE8e-797 with SmuCas9, an Nme2Cas9 ortholog from *Simonsiella muelleri ATCC 29453* [37], or introduced point mutations of eNme2-C [18] in Nme2ABE8e-797.

Moreover, our results indicated that Nme2ABE8e-797 can efficiently correct the *LMNA* c.1824 C > T mutation in Neuro-2a cells. Moreover, mice with efficient single-base substitutions at *TYR* gene loci were successfully generated by using the improved Nme2ABE8e-797.

Overall, we have developed inlaid Nme2ABEs with increased editing activity, wide editing windows, and the ability to target a single-nucleotide PAM, which can provide additional options for therapeutic applications.

## Results

### Design and characterization of Nme2Cas9 adenine base editors with internally inlaid adenosine deaminase

**Fig. 1:**
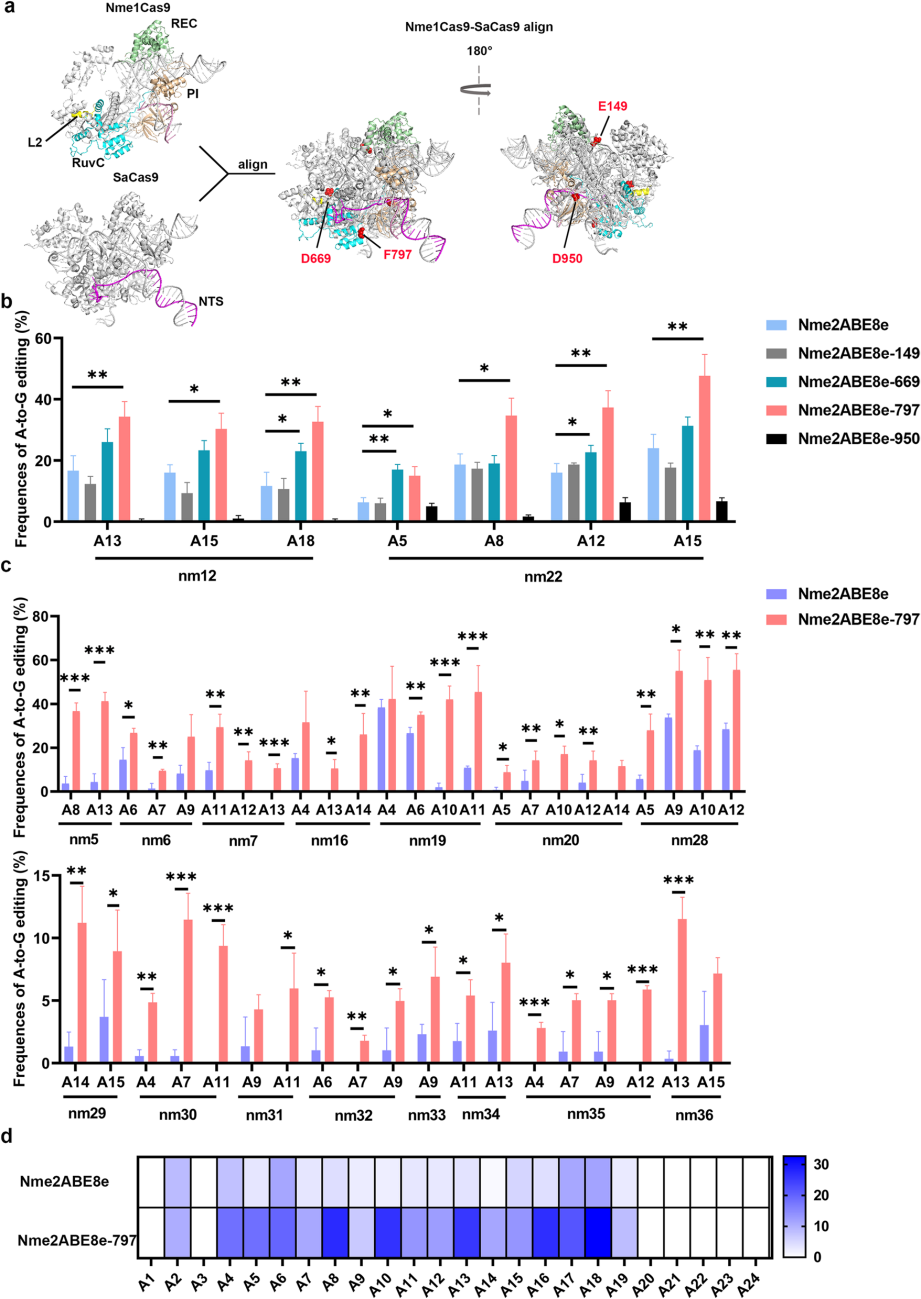
Inlaying TadA8e into the Nme2Cas9 domain can enhance on-target DNA editing in HEK293T cells. **a** Cartoon representations of the structure Nme1Cas9/sgRNA/DNA ternary complex structure (PDB: 6KC7), SaCas9/sgRNA/DNA ternary complex (PDB:7VW3) and alignment of Nme1Cas9/SaCas9 complex. Nme2Cas9 is 98% identical to Nme2Cas9 outside the WED and PAM-interacting domains. Deaminase domain insertion sites (Nme2Cas9 aa numbers) are shown as red spheres. **b** Comparison of A-to-G editing efficiency produced by Nme2ABE8e and four inlaid NmeABE8es using Sanger sequencing at 2 endogenous human genomic loci. Bars represent mean values, and error bars represent the s.d. values of three independent biological replicates, with * and **representing *P* < 0.05 and 0.01, respectively (two-tailed unpaired t test). **c** Comparison of A-to-G editing efficiency produced by Nme2ABE8e and Nme2ABE8e-797 using next-generation sequencing at 15 endogenous human genomic loci. Bars represent mean values, and error bars represent the s.d. of three independent biological replicates, with *, ** and *** representing *P* < 0.05, 0.01 and 0.001, respectively (two-tailed unpaired t test). **d** Heatmap showing the average editing efficiency of and editing windows of Nme2ABE8e and Nme2ABE8e-797 at each position across 26 sites, each of which was independently repeated three times

Based on protein secondary structure, we hypothesized that inlaying TadA8e into the Nme2Cas9 domain to bring the deaminase closer to its target site might improve editing efficiency (Fig. 1a, Additional file 1: S1a). Due to the absence of data on the full-length nontarget strand (NTS) within the current structure of the Nme2Cas9-sgRNA-dsDNA ternary complex [35], we compared the structures of Nme1Cas9 (PDB:6KC7), Nme2Cas9 (PDB:6JE3) [35] and SaCas9 (PDB:7VW3) [30] to simulate the position of the NTS in the Nme2Cas9 complex (Fig. 1a).

Four Nme2ABEs were constructed under the guidance of the structural information. The evolved *Escherichia coli* tRNA-specific adenosine deaminase (TadA8e) was inserted between amino acids 149 and 150, 669 and 670, 797 and 798, and 950 and 951 of the Nme2Cas9 protein (Fig. 1a, Additional file 1: Fig. S1a and b). These positions were chosen due to their locations in the unstructured loops of the Nme1Cas9 (PDB:6KC7) and Nme2Cas9 complex (PDB:6JE3) [35] (Fig. 1a). Notably, we found that residue 797 in the loop of Nme2Cas9 was conformationally analogous to residue 745 and 1058 in the poorly crystallized protein loops of SaCas9 and SpCas9, respectively [17, 29, 30]. The loop of residue 669 in the disordered region of Nme2Cas9 is adjacent to the L2 linker, which connects the RuvC domain, which relocates to approach the nontarget strand when Cas9 is in the active state [5, 35]. The resulting inlaid base editors were identified as Nme2ABE8e-149, Nme2ABE8e-669, Nme2ABE8e-797 and Nme2ABE8e-950 based on their respective inlaid positions (Additional file 1: Fig. S1b).

First, the editing activity of these inlaid base editors was evaluated at 2 endogenous sites in human HEK293T cells (Fig. S1a, Additional file 1: Table S1). Sanger sequencing results revealed that Nme2ABE8e-669 and Nme2ABE8e-797 resulted in 1.7- and 2.3-fold higher editing efficiencies than Nme2ABE8e, respectively (Fig. 1b). Furthermore, the average editing efficiency mediated by Nme2ABE8e-797 (28.9%) was higher than that mediated by Nme2ABE8e-669 (21.1%) (Fig. 1b). Therefore, all subsequent experiments were performed exclusively with Nme2ABE8e-797.

Subsequently, the activity and editing window of Nme2ABE8e-797 were characterized against 26 endogenous sites in mammalian cells, including HEK293T and Neuro-2a cells (Additional file 1: Table S1). Nme2ABE8e-797 improved overall on-target editing efficiencies compared to its Nme2ABE8e counterpart (Fig. 1c, d, Additional file 1: Fig. S2 and S3) and achieved up to a 3.2-fold increase in editing efficiency at the A9-A15 positions compared with Nme2ABE8e (Fig. 1c, d, Additional file 1: Fig. S2 and S3). In addition, Nme2ABE8e-797 outperformed Nme2ABE8e by up to 2.8-fold at the A17 and A18 positions near the PAM (Fig. 1d). Moreover, Nme2ABE8e-797 exhibits enhanced editing capabilities at 9 of 26 sites that are difficult to edit using Nme2ABE8e in both HEK293T and Neuro-2a cells (Fig. 1c, Additional file 1: Fig. S2 and S3).

Overall, Nme2ABE8e-797 vastly increases editing efficiency, can efficiently edit sites that were challenging for Nme2ABE8e, and displays a wide editing activity window spanning adenine positions 4 to 18.

### *Chimeric Nme2ABE8e-797*^*Smu*^* or evolved Nme2ABE8e-797*^*−C*^* expands the PAMs of Nme2ABE*

**Fig. 2:**
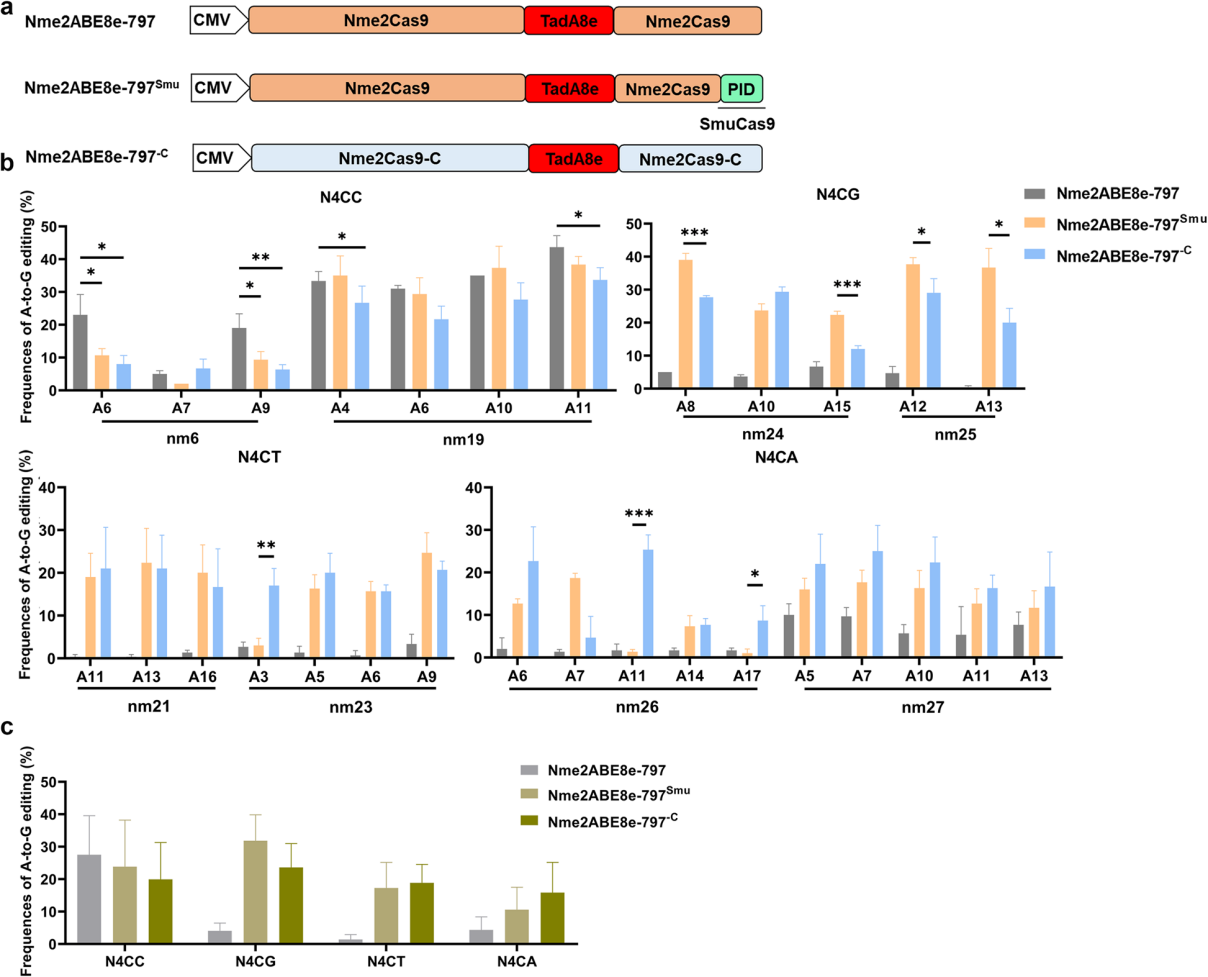
SmuCas9 PAM-interacting domain chimeras or evolved Nme2Cas9 variants expand the targeting scope of Nme2ABE8e-797. **a** Schematic diagram of Nme2ABE8e-797, Nme2ABE8e^−Smu^ and Nme2ABE8e^−C^. **b** Comparison of A-to-G editing efficiency produced by Nme2ABE8e-797, Nme2ABE8e-Smu and Nme2ABE8e-C using Sanger sequencing at 8 PAM-matched N4CN sites in HEK293T cells. Bars represent mean values, and error bars represent the s.d. values of three independent biological replicates, with *, ** and *** representing *P* < 0.05, 0.01 and 0.001, respectively (two-tailed unpaired t test). **c** Data from (**b**) were aggregated and replotted. Comparison of average A-to-G editing efficiency produced by Nme2ABE8e^−Smu^ and Nme2ABE8e^−C^ in HEK293T cells. Bars represent mean values, and error bars represent the s.d. of three independent biological replicates

The activity of BEs were limited to editing in specific editing windows specified by the distance from the PAM. Here, we sought to increase the targeting scope of Nme2ABEs by altering their PAM recognition properties. Replacing the PAM-interacting domain of Nme2ABE8e-797 with that of SmuCas9 [37] or introducing point mutations of eNme2-C into Nme2ABE8e-797 [18] resulted in two modified versions of Nme2ABE, Nme2ABE8e-797^Smu^ and Nme2ABE8e-797^−C^, respectively (Fig. 2a). A panel of two N4CC and six N4CD (D = A, G or T) PAM targets was used to test the activity of Nme2ABE8e-797^Smu^ and Nme2ABE8e-797^−C^ in HEK293T cells (Additional file 1: Table S1). Sanger sequencing results revealed that both Nme2ABE8e-797^Smu^ and Nme2ABE8e-797^−C^ displayed efficient editing at all N4CN PAM sites, corresponding to a 6.0-fold and a 5.9-fold average improvement in activity at N4CD PAM sites over Nme2ABE8e-797, respectively (Fig. 2b, c). Nme2ABE8e-797^Smu^ exhibited higher editing activities for N4CG PAMs (31.9% and 23.6% average A•T-to-G•C conversion for Nme2ABE8e-797^Smu^ and Nme2ABE8e-797^−C^, respectively), but there was a slight decrease in editing activity for N4CW (W = A or T) PAMs compared with Nme2ABE8e-797^−C^ (13.9% and 17.8% average A•T-to-G•C conversion for Nme2ABE8e-797^Smu^ and Nme2ABE8e-797^−C^, respectively). In addition, Nme2ABE8e-797 exhibited a higher average editing efficiency than both Nme2ABE8e-797^Smu^ and Nme2ABE8e-797^−C^ for N4CC PAM sites (27.1%, 23.1% and 18.7% average A•T-to-G•C conversion for Nme2ABE8e-797, Nme2ABE8e-797^Smu^ and Nme2ABE8e-797^−C^, respectively) (Fig. 2b, c).

PAM-broadened Cas9 variants have been shown to increase off-target activity due to the increased number of sequences recognized as PAMs [28, 31, 36]. To evaluate Cas9-dependent off-target activity, two protospacer sites (nm12 and nm19) were selected. The six potential off-target sites (POTs) were predicted to analyze site-specific edits according to Cas-OFFinder [4] (Additional file 1: Table S4). For 4 of the 6 predicted sites, next-generation sequencing showed that NmeABE8e-797, Nme2ABE8e-797^Smu^ and Nme2ABE8e-797^−C^ did not generate any Cas9-dependent off-target base editing (Additional file 1: Fig. S4a), which is consistent with previous reports [18]. A recently reported sensitive and cost-effective orthogonal R-loop assay was used to assess the Cas9-independent OT effect on the SaCas9-induced R-loop region, including Sa site 3 and site 4, which had been detected in previously reported studies [27, 36]. Similarly, NmeABE8e-797, Nme2ABE8e-797^Smu^ and Nme2ABE8e-797^−C^ did not show significantly increased Cas9-independent OT activities compared with Nme2ABE8e (Additional file 1: Fig. S4b). These results suggested that Nme2ABE8e-797^Smu^ and Nme2ABE8e-797^−C^ can target and install precise editing at sites with N4CN PAMs.

### Application of Nme2ABE8e-797 to disease-relevant loci in Neuro-2a cells and gene-edited mice

**Fig. 3:**
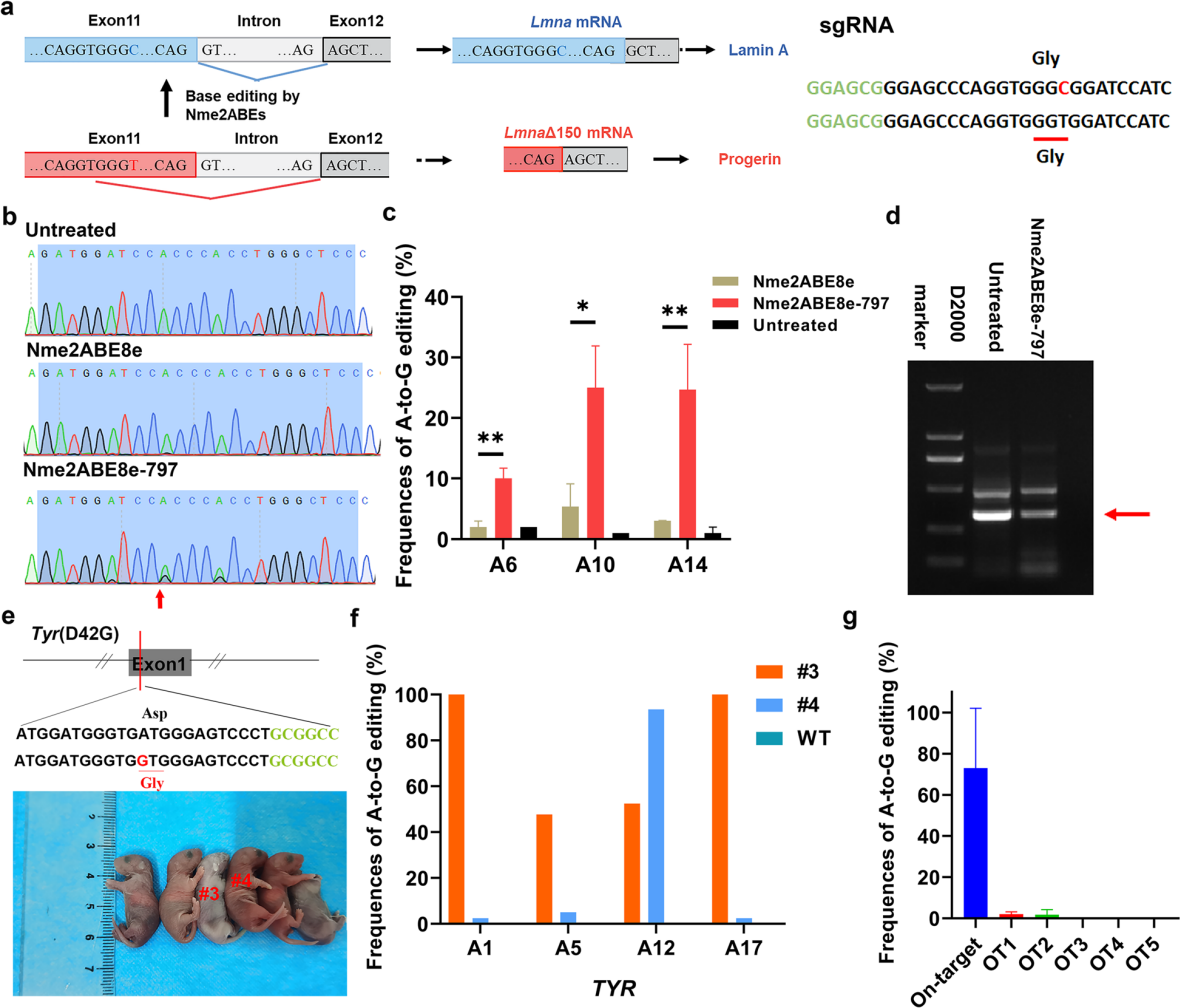
Application of Nme2ABE8e-797 to disease-relevant loci in Neuro-2a cells and mice. **a** Schematic representation of the strategy that restores the in-frame deletion of 150 nt (LMNA Δ150) of the *LMNA* transcript (left). The target sequence for reverting the *LMNA* c.1824 C > T mutation in mouse Neuro-2a *LMNA* c.1824 C > T mutation cell lines is shown. The PAM-targeted and sgRNA-targeted sequences are shown in green and black, respectively. The substituted bases are indicated in red (right). **b** Representative Sanger sequencing chromatograms of the *LMNA* locus. The red arrow indicates the substituted nucleotide. **c** Comparison of A-to-G editing efficiency produced by Nme2ABE8e and Nme2ABE8e-797 using Sanger sequencing in mouse Neuro-2a *LMNA* c.1824 C > T mutation cell lines. Bars represent mean values, and error bars represent the s.d. of three independent biological replicates, with * and ** representing *P* < 0.05 and 0.01, respectively (two-tailed unpaired t test). **d** RT‒PCR analysis of mis-spliced *LMNA* mRNA. The red arrow indicates the mis-spliced *LMNA* mRNA. **e** The target sequence at the *TYR* (p.D42G) locus. The PAM-targeted and sgRNA-targeted sequences are shown in green and black, respectively. The substituted bases are marked in red. **f** Characterization of the targeted modifications in *TYR* (p.D42G) mice using next-generation sequencing. **g** Analysis of off-target effects in *TYR* mutant mice using next-generation sequencing. Data are presented as mean ± s.d. (*n* = 2 mice)

A point mutation (c.1824 C > T; p.G608G) in the Lamin A (*LMNA*) gene, leading to a mis-splicing event that results in the loss of 50 amino acids from the Lamin A protein (Fig. 3a), which is characterized by Hutchinson–Gilford progeria syndrome (HGPS or progeria) [9, 13, 15].

To determine whether the domain-inlaid Nme2-ABE could restore c.1824 C > T in *LMNA*, Nme2ABE8e and Nme2ABE8e-797 together with a corresponding sgRNA targeting the N4CC PAM were cotransfected into mouse Neuro-2a *LMNA* c.1824 C > T mutation cell lines, respectively (Fig. 3a). Sanger sequencing revealed that Nme2ABE8e-797 resulted in 4.7-fold higher editing than Nme2ABE8e, while no significant base editing events were observed by using Nme2ABE8e (Fig. 3b, c). In addition, correction of the progeria mutation was previously demonstrated with a PAM-expanded ABE7.10max-VRQR [24]. Sanger sequencing revealed that Nme2ABE8e-797 resulted in higher editing efficiency than ABE7.10max-VRQR, although the SpCas9-derived ABE did not show any bystander editing (Additional file 1: Fig. S5). Consistent with genomic correction of *LMNA* c.1824 C > T, we observed a reduction in the levels of mis-spliced *LMNA* mRNA using Nme2ABE8e-797 (Fig. 3d). These results indicated that the inlaid Nme2ABE facilitates more efficient correction of the HGPS mutation at the target locus in Neuro-2a cells.

We next investigated whether inlaid Nme2ABE can be used to mimic the disease-relevant point mutations of the *TYR* mutation (p.D42G), which is the major causal genetic mutation responsible for human ocular albinism (OA) and oculocutaneous albinism (OCA) [32] in mice. Nme2ABE8e-797 mRNA and a single sgRNA that targeted the *TYR* gene were coinjected into the cytoplasm of zygotes. Then, 2-cell embryos were transferred to recipient female mice. As shown in Fig. 3e and f, two of the six desired *TYR* (p.D42G) mutations were identified in mouse pups by using next-generation sequencing, and the results showed 52.46% and 93.57% *TYR* mutation efficiencies in pups #3 and #4, respectively. Furthermore, the five potential off-target sites (POTs) with up to 4-nucleotide mismatches in the mouse genome for sgRNA were predicted to analyze site-specific edits according to Cas-OFFinder [4] (Additional file 1: Table S4). No undesirable base changes or sgRNA sequence-dependent off-target mutations were observed in these mice by next-generation sequencing, indicating the efficiency and precision of Nme2ABE8e-797 in mice (Fig. 3g).

Finally, to test the in vivo therapeutic potential of the single-AAV inlaid Nme2ABEs system, we attempted to install mutations in Neuro-2a cells that are associated with a decreased risk of cardiovascular disease in humans [7, 10, 34]. Accordingly, improved AAV expression cassettes [8] were designed with both ABE expression and guide RNA targeting the exon 6 splice donor of mouse *PCSK9* (Additional file 1: Fig. S6a). The editing activity was assessed by transfection of these single-AAV plasmids encoding Nme2ABE8e-797^−C^ and sgRNA targeting sites with N4CT PAM in Neuro-2a cells. The efficiency of base editing was determined to range from undetectable to 25%, as measured by Sanger sequencing (Additional file 1: Fig. S6b, c), which indicates that Nme2ABE8e-797^−C^ may enable in vivo therapeutic applications with single-AAV delivery in the future.

Collectively, these results demonstrated that the improved domain-inlaid Nme2ABE8es can effectively restore or install disease-relevant loci in Neuro-2a cells and mice.

## Discussion

In this study, an improved Nme2ABE was developed, which is embedded with a structure domain, possessing greater editing activity and a wide editing window while targeting a single nucleotide PAM. First, Nme2ABE8e-797 has been shown to vastly augment editing efficiency with a wide editing activity window spanning adenine positions 4 to 18. More importantly, Nme2ABE8e-797 can efficiently edit some loci that were previously challenging for Nme2ABE8e. In addition, we engineered two modified versions of Nme2ABEs, Nme2ABE8e-797^Smu^ and Nme2ABE8e-797^−C^, which can precisely target and install editing at sites with N4CN PAMs. Moreover, our findings proved that domain-inlaid Nme2ABE8es can be applied to restore or install disease-relevant loci in Neuro-2a cells and mice, highlighting their potential for single-AAV delivery for in vivo therapeutic applications.

Previous studies have demonstrated that ABE, using ABEmax architecture [23] with a series of compact Cas9s, had limited compatibility [1, 19, 26]. In contrast, recent studies have shown that TadA-8e, developed through phage-assisted evolution, has improved compatibility and activity with compact Cas9s such as Nme2Cas9, CjeCas9 and SauriCas9 [8]. However, the editing efficiencies of compact Cas9-TadA-8es are substantially lower than those of the corresponding CBEs. More importantly, certain sites cannot be effectively edited by these compact Cas9s [8]. This limitation can be attributed to the stronger binding affinity of these small and compact Cas9s compared to SpCas9, as well as the limited accessibility of deaminases to the nontarget strand [3]. To overcome this limitation, the development of domain-inlaid editors is essential and will further enhance the applicability of compact ABEs, including miniBEs such as IscB [2] and TnpB [21]. However, the current understanding of the structure of Cas9-sgRNA-dsDNA ternary complexes lacks data regarding the full-length nontarget strand, which complicates the design of domain insertions by this approach. Therefore, further investigation is required to better understand nontarget strand recognition during Cas9-guided editing.

The target slope of these compact base editors was further expanded by our design of Nme2ABE8e-797^Smu^ and Nme2ABE8e-797^−C^, which identifies single-cytidine PAMs.

Although our Nme2ABE8e-797^Smu^ and Nme2ABE8e-797^−C^ had detectable activity at all N4CN PAM target sites tested, we observed a reduction in activity at sites with N4CC PAMs when compared to domain-inlaid Nme2ABE8e-797. Nme2ABE8e-797 is recommended for target sites carrying N4CC PAMs, Nme2ABE8e-797^Smu^ for N4CG PAMs and Nme2ABE8e-797^−C^ for N4CW PAMs (W = A or T). Additionally, the recently developed eNme2-T.1 and eNme2-T.2 variants [18] can access N4TN PAM sequences with comparable editing efficiencies, thereby increasing the usability of inlaid-domain Nme2ABE. Furthermore, our findings indicate that engineered PAM-interacting domain variants facilitate effective editing at N4CN PAMs compared to the evolved Nme2ABE8e-797^−C^, which implies that numerous mutations outside the PAM-interacting domain could contribute to improved activity. As a result, future work may focus on developing engineered Nme2Cas9 PAM-interacting domain variants that further relax the 5th position of the PAM, similar to the SpRYCas9 variant [36].

## Conclusions

Overall, in light of the increased on-target DNA editing, minimal PAM, wide editing window, and compact size of the inlaid-domain Nme2ABEs, we anticipate that these tools will lead to the development of novel treatments for genetic diseases and cancers.

## Methods

### Plasmid construction

To construct the Nme2ABE8e plasmid, the reading frame encoding TadA-8e from ABE8e (Addgene:#138,489) [33] was amplified by PCR and used to replace the TadA-8.17 fragment within Nme2ABE8.17 [39] using a ClonExpress Ultra One Step Cloning Kit (Vazyme, Nanjing, China). TadA-8e DNA fragments were then synthesized and cloned and inserted into different positions of Nme2ABE8e by GenScript Biotech (Nanjing, China). The sgRNA plasmid was constructed and inserted into the PUC57 and 74,707 vectors and transformed into *Escherichia coli* (DH5α) (Sangon Biotech, Shanghai, China). Spacer oligos and sgRNA scaffold oligos were synthesized and cloned and inserted into the 74,707 and pUC57-sgRNA expression vectors. The sequences of the spacer oligos are listed in Additional file 1: Table S1.

### Cell culture and transfection

Human kidney epithelial cells (HEK293T) and mouse neuroblastoma N2a cells (Neuro-2a) were cultured in Dulbecco's modified Eagle's medium (DMEM) (Meilun Biotechnology Co., Ltd) supplemented with 10% fetal bovine serum (HyClone), 2 mM GlutaMAX (Life Technologies), 100 U/ml penicillin, and 100 mg/ml streptomycin and incubated at 37 °C in an atmosphere containing 5% CO_2_. The cells were seeded into 12-well plates (Jet, Guangzhou, China) at a concentration of ~ 2 × 10^5^ cells per well in 1 mL of complete growth medium. Transfections were performed when the cell density reached approximately 80%-90% confluence. Subsequently, 3 µL of Hieff TransTM Liposome nucleic acid transfection reagent (Yeasen Biotechnology, Shanghai, China) with 0.5 μg CRISPR base editor plasmid and 0.5 µg sgRNA-expressing plasmid was transfected into cells 12–16 h after plating. After 72 h, the cells were washed twice with phosphate-buffered saline (PBS) before lysis using a One Step Mouse Genotyping Kit (Vazyme Biotech, China) at 55 °C for 25 min, followed by enzyme inactivation at 95 °C for 5 min. Cell genomic DNA was then used as a template for polymerase chain reaction (PCR) amplification.

### Targeted PCR amplification

Targeted sites were amplified from genomic DNA using 2X M5 HiPer plus Taq HiFi PCR mix (with blue dye) V. 2 (Mei5 Biotechnology Co., Ltd). Briefly, PCR was performed in a volume of 25 µL comprising 12.5 µL of 2X M5 HiPer plus Taq HiFi PCR mix, 1 µl of forward and reverse primers, and 1 µl of cell genomic DNA under thermocycling conditions of 95 °C initial denaturation for 5 min and 38 cycles of 95 °C denaturation for 30 s, 58 °C annealing for 30 s, and 72 °C extension for 30 s with a 72 °C final extension for 5 min. Sanger sequencing of PCR amplicons was performed by Sangon Biotech (Shanghai, China). The primer sequences are listed in Additional file 1: Table S2.

### In vitro* transcription*

The plasmid of Nme2ABE8e-797 was linearized with NotI and transcribed in vitro using the HiScribe™ T7 ARCA mRNA kit (NEB). These mRNAs were purified using the RNeasy Mini Kit (Qiagen, Hilden, Germany) in accordance with the manufacturer’s protocol. The sgRNA oligos were annealed into pUC57-sgRNA expression vectors containing a T7 promoter. The sgRNAs were then amplified and transcribed in vitro using a MAXIscript T7 kit (Ambion; Applied Biosystems, CA, USA) and purified using a miRNeasy Mini Kit (Qiagen, Hilden, Germany) following the manufacturer’s protocol.

### Microinjection and embryo transfer

ABE mRNA (60 ng/µl) and sgRNA (30 ng/µl) were cotransfected into the cytoplasm of pronuclear-stage embryos. The injected embryos were cultured at 37 °C under 5% CO2 in air until the two-cell stage, after which approximately 30–50 injected embryos were transferred into the oviduct of the recipient mother.

### Mouse genotyping

Newborn mouse toes were clipped for genomic DNA extraction. The genomic regions surrounding the target site were PCR amplified and then subjected to next-generation sequencing. Targeted sites were amplified from genomic DNA using 2X M5 HiPer plus Taq HiFi PCR mix (with blue dye) V. 2 (Mei5 Biotechnology Co., Ltd). The primers used for genotyping are listed in Additional file 1: Table S3.

### Estimation of editing frequency

EditR (https://moriaritylab.shinyapps.io/editr_v10/) online software [22] was applied to estimate the editing frequency of Sanger sequencing.

### Off-target assay

The potential Cas9-dependent off-target sites (POTs) with up to 4-nucleotide or 5-nucleotide mismatches in the mouse genome or human genome for sgRNA were predicted to analyze site-specific edits in accordance with Cas-OFFinder [4] (http://www.rgenome.net/cas-offinder/). An orthogonal R-loop assay to evaluate Cas9-independent off-target effects was performed as previously described [11]. The genomic regions surrounding the off-target site were PCR amplified and then subjected to next-generation sequencing. All primers for the off-target assay are listed in Additional file 1: Table S3.

### ddddNext-generation sequencing

The genomic regions surrounding the on- and off-target sites were PCR amplified using 2X M5 HiPer plus Taq HiFi PCR mix (with blue dye) V. 2 (Mei5 Biotechnology Co., Ltd). Primers carrying the bridging sequence used to amplify the flanking region of each on- and off-target site are listed in Additional file 1: Table S3. Samples were sequenced commercially using the high-throughput tracking of mutations (Hi-TOM) method [25]. The sequencing results were analyzed using the Hi-TOM online tool (https://www.hi-tom.net/hi-tom/index-CH.php) [25].

### Statistical analyses

All data are expressed as the mean ± SD, with at least three individual determinations carried out in all experiments. The data were analyzed with a two-tailed unpaired *t* test using GraphPad Prism software 8.0. *p* < 0.05 indicated statistical significance (*p < 0.05, **p < 0.01, ***p < 0.001).

### Supplementary Information

**Additional file 1:** **Fig. S1.** Flow diagram for designing and assessing inlaid-Nme2ABEs.** Fig. S2. **Comparison of A-to-G editing efficiency produced by Nme2ABE8e and inlaid NmeABE8es at 4 endogenous loci using Sanger sequencing in HEK293T cells. **Fig. S3. **Comparison of A-to-G editing efficiency produced by Nme2ABE8e and NmeABE8e-797 at 5 endogenous loci using Sanger sequencing in Neuro-2a cells.** Fig. S4.** Off-target analyses of inlaid-Nme2ABE8es.** Fig. S5. **Nme2ABE8e-797 mediate higher A•T to G•C conversion than Sp-ABE-VRQR in mouse Neuro-2a LMNA c.1824 C>T mutation cell lines.** Fig. S6. **Nme2ABE8e-797^-C^ enable efficient editing in Neuro-2a cells.** Table S1. **Target sites in mammalian cells used in this study. **Table S2. **Primers used to amplify genomic DNA for Sanger sequencing in this study. **Table S3.** Primers used to amplify genomic DNA for Next generation sequencing in this study. **Table S4.** The potential off-target sites (POTS) used in this study. **Supplementary Notes** Nucleotide sequence of Nme2ABE8e-797, Nme2ABE8e-797^Smu^ and Nme2ABE8e-797^-C^ base editors described in this manuscript.

**Additional file 2.** All data generated or analyzed during this study are included in this published article.

**Additional file 3.** Raw Images for Fig. 3d.

## Data Availability

All data generated or analysed during this study are included in this published article and its supplementary information files (Additional files 2 and 3).

## Abbreviations

PAM: Protospacer-adjacent motif
ABEs: Adenine base editors
TadA: TRNA-specific adenosine deaminase
DSB: Double-stranded DNA breaks
HGPS: Hutchinson–Gilford progeria syndrome
OA: Ocular albinism
OCA: Oculocutaneous albinism
sgRNA: Single-guide RNA
