# Supplementary material for: Engineered domain-inlaid Nme2Cas9 adenine base editors with increased on-target DNA editing and targeting scope
Source: BMC Biol. 2023 Nov 9;21:250. doi: 10.1186/s12915-023-01754-4 (PMC10636962; doi:10.1186/s12915-023-01754-4)
Supplement: Supplementary file 1 — Additional file 1: Fig. S1. Flow diagram for designing and assessing inlaid-Nme2ABEs. Fig. S2. Comparison of A-to-G editing efficiency produced by Nme2ABE8e and inlaid NmeABE8es at 4 endogenous loci using Sanger sequencing in HEK293T cells. Fig. S3. Comparison of A-to-G editing efficiency produced by Nme2ABE8e and NmeABE8e-797 at 5 endogenous loci using Sanger sequencing in Neuro-2a cells. Fig. S4. Off-target analyses of inlaid-Nme2ABE8es. Fig. S5. Nme2ABE8e-797 mediate higher A•T to G•C conversion than Sp-ABE-VRQR in mouse Neuro-2a LMNA c.1824 C>T mutation cell lines. Fig. S6. Nme2ABE8e-797-C enable efficient editing in Neuro-2a cells. Table S1. Target sites in mammalian cells used in this study. Table S2. Primers used to amplify genomic DNA for Sanger sequencing in this study. Table S3. Primers used to amplify genomic DNA for Next generation sequencing in this study. Table S4. The potential off-target sites (POTS) used in this study. Supplementary Notes Nucleotide sequence of Nme2ABE8e-797, Nme2ABE8e-797Smu and Nme2ABE8e-797-C base editors described in this manuscript. [file 12915_2023_1754_MOESM1_ESM.docx]

**SUPPLEMENTARY INFORMATION**

**
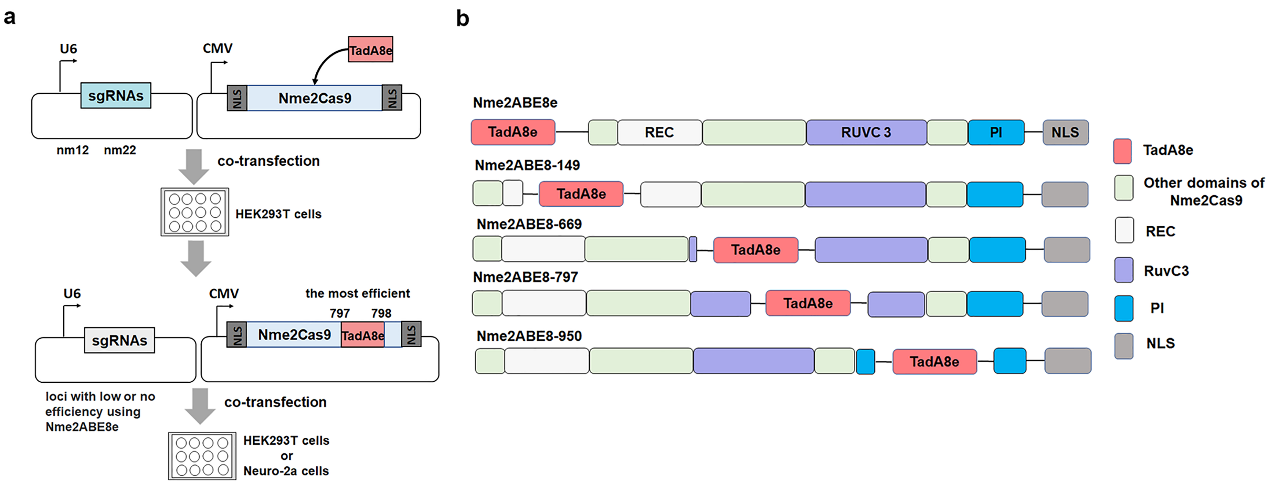
**

**Fig. S1.** Flow diagram for designing and assessing inlaid-Nme2ABEs.

**a.** Flow diagram for designing and assessing inlaid-Nme2ABEs in mammal cells. NLS, bipartite nuclear localization signal.

**b.** Cartoon representations showing the architectures of Nme2ABE8e and inlaid NmeABE8es (Nme2ABE8e-149, Nme2ABE8e-669, Nme2ABE8e-797, Nme2ABE8e-950). NLS, bipartite nuclear localization signal.

**
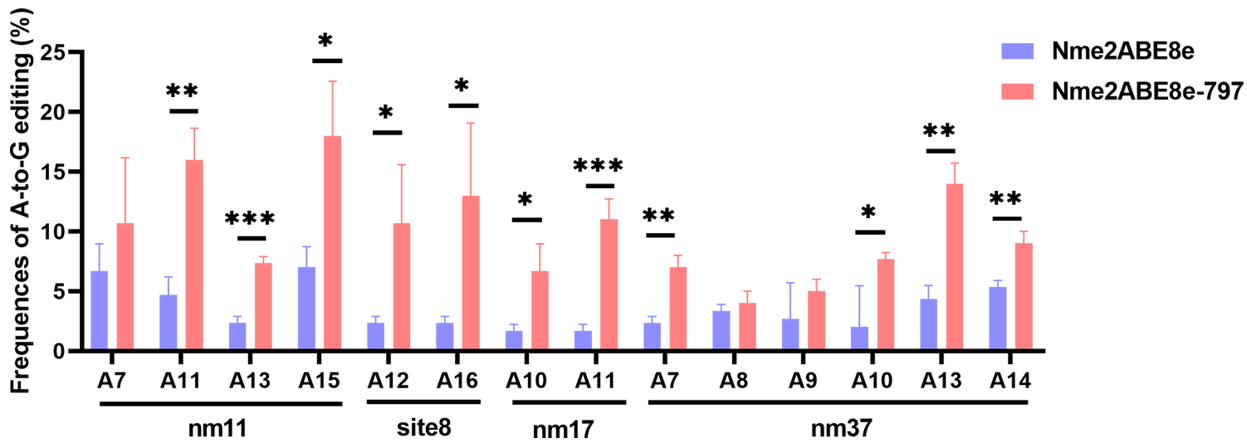
**

**Fig. S2****.** Comparison of A-to-G editing efficiency produced by Nme2ABE8e and inlaid NmeABE8es at 4 endogenous loci using Sanger sequencing in HEK293T cells. Bars represent mean values, and error bars represent the s.d. of three independent biological replicates, with *, ** and *** representing P < 0.05, 0.01 and 0.001, respectively (two- tailed unpaired t test).


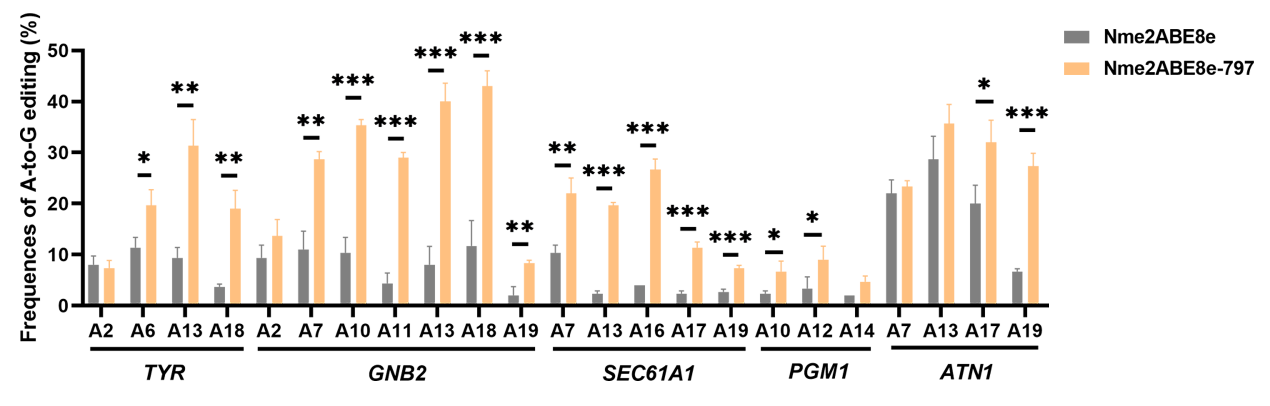


**Fig. S3.** Comparison of A-to-G editing efficiency produced by Nme2ABE8e and NmeABE8e-797 at 5 endogenous loci using Sanger sequencing in Neuro-2a cells. Bars represent mean values, and error bars represent the s.d. of three independent biological replicates, with *, ** and *** representing P < 0.05, 0.01 and 0.001, respectively (two- tailed unpaired t test).


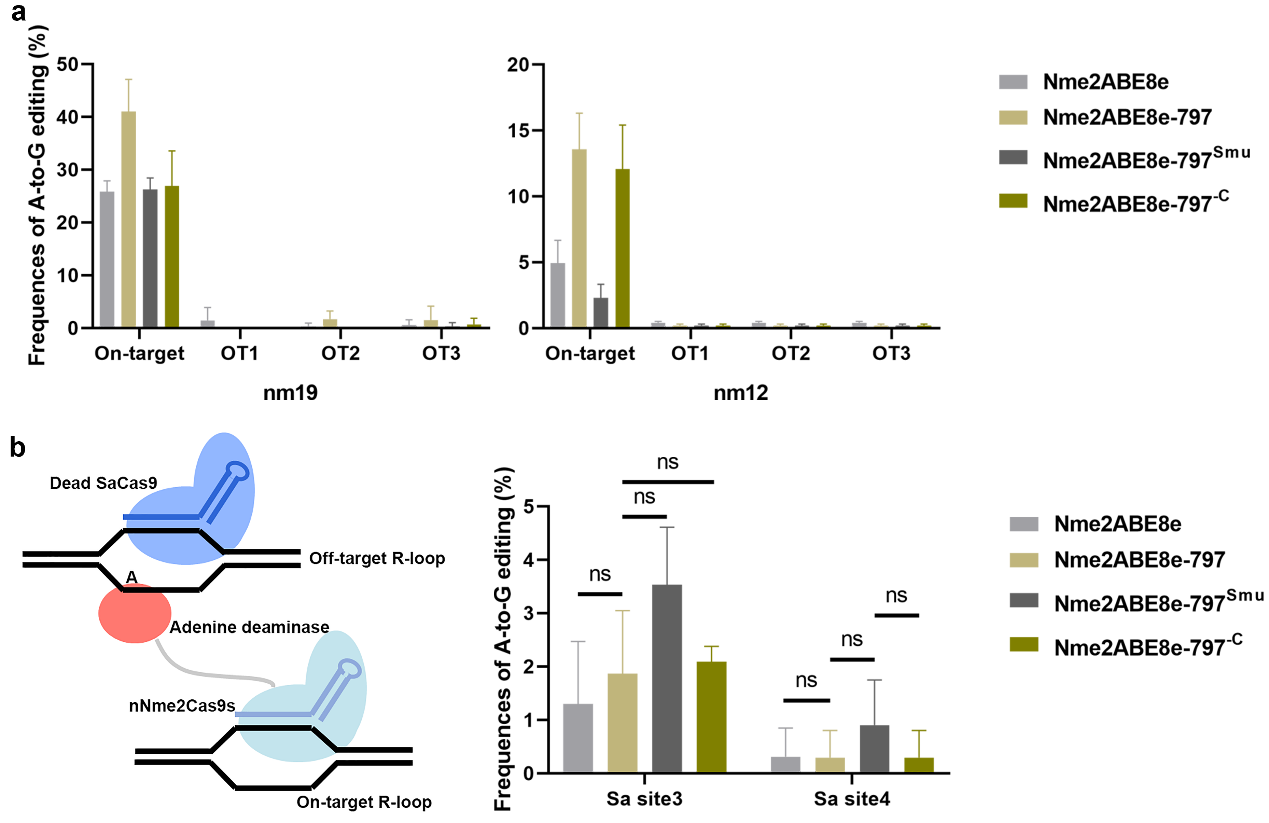


**Fig. S4.** Off-target analyses of inlaid-Nme2ABE8es.

**a.** Cas9-dependent OT editing frequencies of Nme2ABE8e, Nme2ABE8e-797, Nme2ABE8e-797^Smu^, and Nme2ABE8e-797^-C^ at the nm19 and nm12 off-target sites. Values and error bars reflect the mean ± s.d. and n= 3 biologically independent experiments.

**b.** Cas9-independent OT editing frequencies of Nme2ABE8e, Nme2ABE8e-797, Nme2ABE8e-797^Smu^, and Nme2ABE8e-797^-C^ at Sa site3 and site4. The dSaCas9 together with a gRNA was used to induce a stable ssDNA region (orthogonal R loop) at a specific locus, thus artificially magnifying Cas9-independent deamination. Values and error bars reflect the mean ± s.d. and n= 3 biologically independent experiments.


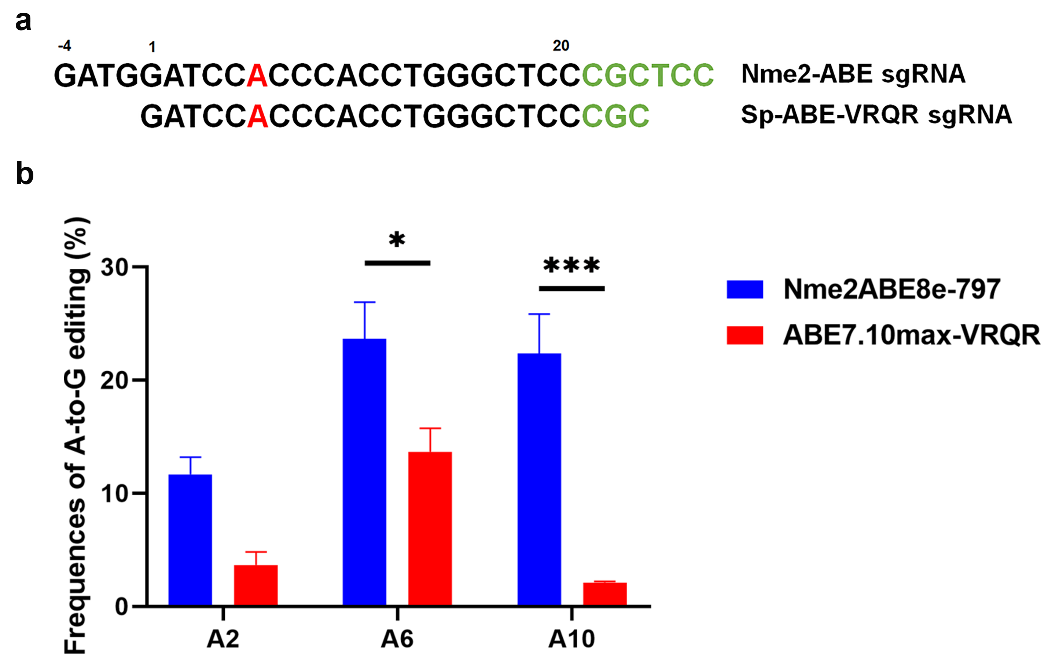


**Fig. S5.** Nme2ABE8e-797 mediate higher A•T to G•C conversion than Sp-ABE-VRQR in mouse Neuro-2a LMNA c.1824 C>T mutation cell lines.

**a.** Schematic of designed target sites with NGCNCC PAM to be compatible with Sp-ABE-VRQR and Nme2 gRNA simultaneously. The PAM is shown in green. The on-targeted A is shown in red.

**b.** Comparison of A-to-G editing efficiency produced by Nme2ABE8e-797 and ABE7.10max-VRQR in mouse Neuro-2a *LMNA* c.1824 C>T mutation cell lines. Bars represent mean values, and error bars represent the s.d. of three independent biological replicates, with * and*** representing P < 0.05 and 0.001 (two- tailed unpaired t test).


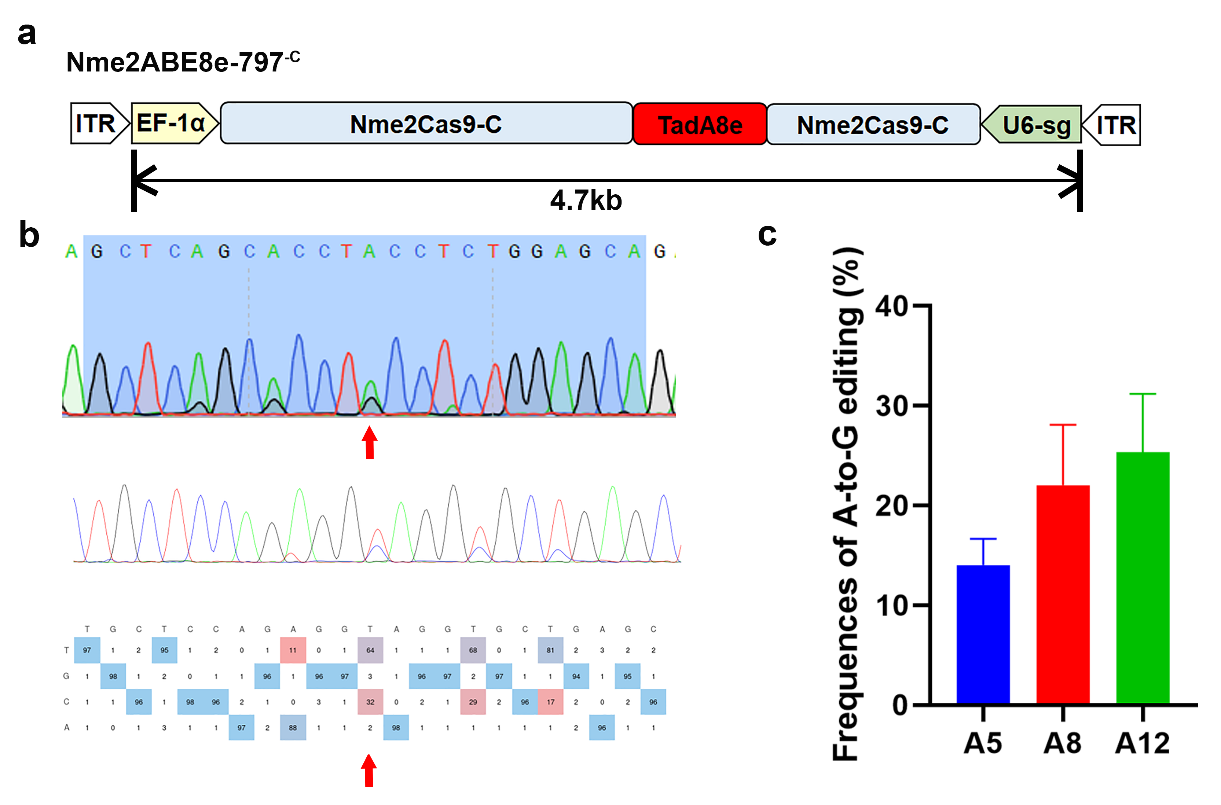


**Fig. S6.** Nme2ABE8e-797^-C^ enable efficient editing in Neuro-2a cells.

**a**. Schematic of the single-AAV Nme2ABE8e-797^-C^ genome.

**b**. Sanger sequencing chromatograms of DNA from *PSCK9* in Neuro-2a cells. The red arrow indicates the substituted nucleotide.

**c**. Base editing activity of Nme2ABE8e-797^-C^ single-AAV plasmid. Bars represent mean values, and error bars represent the s.d. of three independent biological replicates.

| **Table S1. Target sites in mammalian cells used in this study. Target sequence (black), PAM region (green).** | | |
| --- | --- | --- |
| **Target site** | **sequence (5’-3’)** | **PAM** |
| **Nm5** | AACGCCCATGGCAACGGGCAAGT | GCGGCC |
| **Nm6** | GAGGCAAGAGGGCGGCTTTGGGCG | GGGTCC |
| **Nm7** | CCCTGGGAATAAAGAGAAAGCTG | CTCACC |
| **Nm11** | GATTCCAGCCAGACACCCGCCCCC | CGGCCC |
| **Nm12** | TGTGGAACTCTATATTACTTACC | TTATCC |
| **Nm16** | GACCAGCCCCTCGAAGGCAAGGCC | AGGACC |
| **Nm17** | AGCCCCTCGAAGGCAAGGCCAGG | ACCTCC |
| **Nm19** | GTAGATGGAACCGTGGAACGAGG | GAATCC |
| **Nm22** | GAAAATTATGCAGTACTGGGTTG | GGGTCC |
| **Site8** | CGCAAAGCTGCATCCACCCCCCG | AGGACC |
| **Nm21** | GTGGAACTCTATATTACTTACCT | TATCCT |
| **Nm23** | GCACAACCAGTGGAGGCAAGAGG | GCGGCT |
| **Nm24** | GGTGTGCAGACGGCAGTCACTAG | GGGGCG |
| **Nm25** | GTCTCCGCTTTAACCCCCACCTC | CAGCCG |
| **Nm26** | GGTCCAAAGCAGGATGACAGGCA | GGGGCA |
| **Nm27** | GGCTACAGCAACAGGGTGGTGG | ACCTCA |
| **Nm28** | CTAGAGGTAACATGTTGAAGATG | GAATCC |
| **Nm29** | GACCAGCCCCTCGAAGGCAAGGCC | AGGACC |
| **Nm30** | GAGACACTCCAAGAGAGCCTGG | GAATCC |
| **Nm31** | GCAAGAGCACAAGAGGAAGAGAG | AGACCC |
| **Nm32** | GAGGCAAGAGGGCGGCTTTGGGCG | GGGTCC |
| **Nm33** | TATGTTCCAGCTTCCTGGGTCTGC | AGGTCC |
| **Nm34** | GTTTGTCCCCACAGTCCCCAGGGA | AAAGCC |
| **Nm35** | GAGACTATATCACTCTTGGAGCG | GAGGCC |
| **Nm36** | TAGGAAATCTCACAAAAGACCTA | AAGACC |
| **Nm37** | TTCTGGAAAAGGAAGCCCGGACC | CCACCC |
| ***PGM1*** | GCTATCAATATACAGCCGGATGGT | AGCCCC |
| ***TYR*** | ATGGATGGGTGATGGGAGTCCCT | GCGGCC |
| ***SEC61A1*** | CTCTTCATTGCGACTAACATCTGT | GAGACC |
| ***ATN1*** | ACCTTCACCTGCACCAGCAGGATG | CTATCC |
| ***GNB2*** | TACACCACTAACAAGGTAAAGCGG | GGTACC |
| ***PSCK9*** | GCTCAGCACCTACCTCTGGAGCA | GAAGCT |

| **Table S2. Primers used to amplify genomic DNA for Sanger sequencing in this study** | |
| --- | --- |
| **Target site** | **Sequence (5’-3’)** |
| **Nm11** | F TCTTCTCCTGTGGTGGAGT |
|  | R CTGTGCCTCAACCTCCTAAC |
| **Nm12** | F AGAAGGTAAGTGCATGGTAAGG |
|  | R CCAGATTTCTCAGCCTCTTTCT |
| **Nm17** | F GCACTGTCCTGCCCTAAAT |
|  | R CTTTACCAGGAAGGATGGAAGG |
| **Nm19** | F GCACTGTCCTGCCCTAAAT |
|  | R CTTTACCAGGAAGGATGGAAGG |
| **Nm22** | F CTCCTGCTTGTCCAACTAACT |
|  | R GCACTAGTGAACAGGGTGAA |
| **Site8** | F GACCTGAGAGAAGCCTAAGAGA |
|  | R GCTCTCCGTCCAAAGTCAAA |
| **Nm21** | F AGAAGGTAAGTGCATGGTAAGG |
|  | R CCAGATTTCTCAGCCTCTTTCT |
| **Nm23** | F GGGCCTGGAAGTTCGCTAAT |
|  | R TGGATCGCTTTTCCGAGCTT |
| **Nm24** | F CATTCCCTCTTTAGCCAGAGCCGG |
|  | R CAGATCTATTGGAATCCTGGAGTGACC |
| **Nm25** | F GGCGAGGCAGAGGGTCCAAA |
|  | R CTCCTTCTGGGGCCTTTTTCCC |
| **Nm26** | F GAACCCAGGTAGCCAGAGAC |
|  | R TCCTTTCAACCCGAACGGAG |
| **Nm27** | F GCTCAGAAAAAGGGCCCTGA |
|  | R GAGATTCAGTGTGGTGGGGG |
| **Nm37** | F GCAGGTCAAGGGCTGATAA |
|  | R CCACCCATCCACCCATTT |
| ***PGM1*** | F CTGCCATCTGTCAAGGATCTAAT |
|  | R GTACCTATGCAAAGGGCTTAGT |
| ***TYR*** | F GGGAGTGGTTATATAGGTCTTAGC |
|  | R TCTTCTAATCAAGACTCGCTTCTC |
| ***SEC61A1*** | F AAAGAAGCTGGAGGTTGTAGG |
|  | R ACCATGACGAGGCATATGAAA |
| ***ATN1*** | F TGCCAGAAAGCTGAATGATACT |
|  | R CTTCGCTTCCCTTTCCCTTT |
| ***GNB2*** | F GGGAGAATCTGTCAGTAATTTGGG |
|  | R CCTCTTCTGGCTCCACTCA |
| ***PSCK9*** | F GCCTGGAGTTTATTCGGAAGAG |
|  | R GGACAGAAGAGGGACTGGATAA |

| **Table S3. Primers used to amplify genomic DNA for Next generation sequencing in this study** | |
| --- | --- |
| **Forward, 5’ extension** | GGAGTGAGTACGGTGTGC |
| **Reverse, 5’ extension** | GAGTTGGATGCTGGATGG |
| **Nm5** | F: CCTTCCATCCTTCCTGGTAAA |
|  | R: CGCAAAGCTGCATCCAC |
| **Nm6** | F: ACTACCTACGTCAGCACCT |
|  | R: GGCCTGGAAGTTCGCTAAT |
| **Nm7** | F: CTTGCTTCCTCTAGTTGGGATG |
|  | R: TCTCTTAGGCTTCTCTCAGGTC |
| **Nm12** | F: GAGTTCCACCGCCTTGTTTA |
|  | R: ACCAGATTTCTCAGCCTCTTTC |
| **Nm16** | F: CTCCTGATGCTTAGCCAACT |
|  | R: CGCAAAGCTGCATCCAC |
| **Nm19** | F: CTCCCCAGCCCAAACCGC |
|  | R: CCTTCTCAAGGAGTTGGCTAAG |
| **Nm20** | F: TCTCATGGCCTTTGCAGTATAA |
|  | R: AAACCTTCACTGAGCACCTC |
| **Nm28** | F: GCCAGTACATCAAACTCCCCA |
|  | R: AATTCCTGCAACTTCAAGAGTC |
| **Nm29** | F: ACGCCAACACCCACGATCTG |
|  | R: CTCCCCAGCCCAAACCGC |
| **Nm30** | F: GGGATGCAGCTCGTTACCA |
|  | R: TGCAGACGCTCCAGCAG |
| **Nm31** | F: GCCAGAGACTGGCTCTTAAA |
|  | R: TGAGTGTGGCAGGGACT |
| **Nm32** | F: GGCCTGGAAGTTCGCTAAT |
|  | R: CACCTCATGGAATCCCTTCTG |
| **Nm33** | F: CTGTGGGCTTGCAGGAG |
|  | R: CATCAAAGAGGCGAGGGTAG |
| **Nm34** | F: TCATTAGGGTCCAGGGATGT |
|  | R: GCAGTGTGACTCTCGTTCAATA |
| **Nm12-OT1** | F: CCCTTCAATTCCAACTCCTTCC |
|  | R: CAGAGCACTGGTTATCTGTGTTT |
| **Nm12-OT2** | F: GTGCATAATTATCAGCCTTGTC |
|  | R: GACCTGTAAAGACCTCTTTCTT |
| **Nm12-OT3** | F: ATCTGCCAAGGCAAACTC |
|  | R: CACTCATGTCCAGACTA |
| **Nm19-OT1** | F: GGTGGAGGAGTATTTATCAAGCA |
|  | R: AATCTGGGTGCTCCTGTATTG |
| **Nm19-OT2** | F: CGATCACAGGCTAAGAGGAAAG |
|  | R: AGCAGTGCCAGCAGAAAT |
| **Nm19-OT3** | F: GGGATCATCATGGGCAGATTAC |
|  | R: CAGCAGTGCCAGCAGAAATA |
| ***TYR*** | F: TGTGCCTCCTCTAAGAACTTG |
|  | R: GCACTGGCAGGTCCTATTAT |
| **TYR-OT1** | F: AGGAGTTAGAGGAAGGACTGAA |
|  | R: TCATCACCTTTCCCGGTTTC |
| **TYR-OT2** | F: CTGAGTGACGGGTGTGATAA |
|  | R: ATGAGTGAGGGACTGTAGGTGT |
| **TYR-OT3** | F: CTCACTGGAGATTAGAGAAGTCATC |
|  | R: GACTTCCACAGGAGAGGATTATG |
| **TYR-OT4** | F: GGGACTGGTCCTATGATATCTAATC |
|  | R: CCTCTCCCTAAAGGTGACAAAG |
| **YR-OT5** | F: CTGTCCTCTGCTGTGGTTATATT |
|  | R: CATGGAGCCTGAGTCTTTCTT |

| **Table S4 The potential off-target sites (POTS) used in this study** | | |
| --- | --- | --- |
| **Potential Off Target Site** | **PAM** | **Number of mismatches** |
| TYR-OT1: ATcaATGGGaGAgGGGAGTCCCT | TGGTCC | 4 |
| TYR-OT2: ATGGATGGcTGATGtGAGgCCCa | AGAACC | 4 |
| TYR-OT3: ATGGcTGGGaGATcGGAGgCCCT | ACGGCC | 4 |
| TYR-OT4: ATGGcTGGGTtgTGGGAGTgCCT | GGAGCC | 4 |
| TYR-OT5: ATGGAaGGGTGcTGtGAGTaCCT | GAGACC | 4 |
| Nm12-OT1: CTGTGcAgCTCTATATTACTctCC | ACTACT | 4 |
| Nm12-OT2: tTtTGGgAtTCTATATTAaTTACC | ACTACC | 5 |
| Nm12-OT3: tgGTGGAtCTCTAgATTACTTACt | TCATCA | 5 |
| Nm19-OT1: gGTAaATGGAACaaTGGAACGAGa | AGAGCT | 5 |
| Nm19-OT2: tGcAGgTGGAgCtGTGGAACGAGG | TGAGCC | 5 |
| Nm19-OT3: tGaAGgTGGAgCtGTGGAACGAGG | TGAGCC | 5 |

**Supplementary Notes**

Nucleotide sequence of Nme2ABE8e-797, Nme2ABE8e-797^Smu^ and Nme2ABE8e-797^-C^ base editors described in this manuscript.

Nme2ABE8e-797: NLS, Cas9, linker, TadA8e, linker, Cas9, NLS

CCAAAGAAGAAGCGGAAAGTCGCCGCCTTCAAGCCTAACCCAATCAATTACATCCTGGGACTGGCCATCGGAATCGCATCCGTGGGATGGGCTATGGTGGAGATCGACGAGGAGGAGAATCCTATCCGGCTGATCGATCTGGGCGTGAGAGTGTTTGAGAGGGCCGAGGTGCCAAAGACCGGCGATTCTCTGGCTATGGCCCGGAGACTGGCACGGAGCGTGAGGCGCCTGACACGGAGAAGGGCACACAGGCTGCTGAGGGCACGCCGGCTGCTGAAGAGAGAGGGCGTGCTGCAGGCAGCAGACTTCGATGAGAATGGCCTGATCAAGAGCCTGCCAAACACCCCCTGGCAGCTGAGAGCAGCCGCCCTGGACAGGAAGCTGACACCACTGGAGTGGTCTGCCGTGCTGCTGCACCTGATCAAGCACCGCGGCTACCTGAGCCAGCGGAAGAACGAGGGAGAGACAGCAGACAAGGAGCTGGGCGCCCTGCTGAAGGGAGTGGCCAACAATGCCCACGCCCTGCAGACCGGCGATTTCAGGACACCTGCCGAGCTGGCCCTGAATAAGTTTGAGAAGGAGTCCGGCCACATCAGAAACCAGAGGGGCGACTATAGCCACACCTTCTCCCGCAAGGATCTGCAGGCCGAGCTGATCCTGCTGTTCGAGAAGCAGAAGGAGTTTGGCAATCCACACGTGAGCGGAGGCCTGAAGGAGGGAATCGAGACCCTGCTGATGACACAGAGGCCTGCCCTGTCCGGCGACGCAGTGCAGAAGATGCTGGGACACTGCACCTTCGAGCCTGCAGAGCCAAAGGCCGCCAAGAACACCTACACAGCCGAGCGGTTTATCTGGCTGACAAAGCTGAACAATCTGAGAATCCTGGAGCAGGGATCCGAGAGGCCACTGACCGACACAGAGAGGGCCACCCTGATGGATGAGCCTTACCGGAAGTCTAAGCTGACATATGCCCAGGCCAGAAAGCTGCTGGGCCTGGAGGACACCGCCTTCTTTAAGGGCCTGAGATACGGCAAGGATAATGCCGAGGCCTCCACACTGATGGAGATGAAGGCCTATCACGCCATCTCTCGCGCCCTGGAGAAGGAGGGCCTGAAGGACAAGAAGTCCCCCCTGAACCTGAGCTCCGAGCTGCAGGATGAGATCGGCACCGCCTTCTCTCTGTTTAAGACCGACGAGGATATCACAGGCCGCCTGAAGGACAGGGTGCAGCCTGAGATCCTGGAGGCCCTGCTGAAGCACATCTCTTTCGATAAGTTTGTGCAGATCAGCCTGAAGGCCCTGAGAAGGATCGTGCCACTGATGGAGCAGGGCAAGCGGTACGACGAGGCCTGCGCCGAGATCTACGGCGATCACTATGGCAAGAAGAACACAGAGGAGAAGATCTATCTGCCCCCTATCCCTGCCGACGAGATCAGAAATCCTGTGGTGCTGAGGGCCCTGTCCCAGGCAAGAAAAGTGATCAACGGAGTGGTGCGCCGGTACGGATCTCCAGCCCGGATCCACATCGAGACCGCCAGAGAAGTGGGCAAGAGCTTCAAGGACCGGAAGGAGATCGAGAAGAGACAGGAGGAGAATCGCAAGGATCGGGAGAAGGCCGCCGCCAAGTTTAGGGAGTACTTCCCTAACTTTGTGGGCGAGCCAAAGTCTAAGGACATCCTGAAGCTGCGCCTGTACGAGCAGCAGCACGGCAAGTGTCTGTATAGCGGCAAGGAGATCAATCTGGTGCGGCTGAACGAGAAGGGCTATGTGGAGATCGATCACGCCCTGCCTTTCTCCAGAACCTGGGACGATTCTTTTAACAATAAGGTGCTGGTGCTGGGCAGCGAGAACCAGAATAAGGGCAATCAGACACCATACGAGTATTTCAATGGCAAGGACAACTCCAGGGAGTGGCAGGAGTTCAAGGCCCGCGTGGAGACCTCTAGATTTCCCAGGAGCAAGAAGCAGCGGATCCTGCTGCAGAAGTTCGACGAGGATGGCTTTAAGGAGTGCAACCTGAATGACACCAGATACGTGAACCGGTTCCTGTGCCAGTTTGTGGCCGATCACATCCTGCTGACCGGCAAGGGCAAGAGAAGGGTGTTCGCCTCTAATGGCCAGATCACAAACCTGCTGAGGGGATTTTGGGGACTGAGGAAGGTGCGGGCAGAGAATGACAGACACCACGCACTGGATGCAGTGGTGGTGGCATGCAGCACCGTGGCAATGCAGCAGAAGATCACAAGATTCGTGAGGTATAAGGAGATGAACGCCTTTGACGGCAAGACCATCGATAAGGAGACAGGCAAGGTGCTGCACCAGAAGACCCACTTCCCCCAGCCTTGGGAGTTCTTTGCCCAGGAAGTGATGATCCGGGTGTTCGGCAAGCCAGACGGCAAGCCTGAGTTTAGCGGGGGGTCATCTGAGGTGGAGTTTTCCCACGAGTACTGGATGAGACATGCCCTGACCCTGGCCAAGAGGGCACGGGATGAGAGGGAGGTGCCTGTGGGAGCCGTGCTGGTGCTGAACAATAGAGTGATCGGCGAGGGCTGGAACAGAGCCATCGGCCTGCACGACCCAACAGCCCATGCCGAAATTATGGCCCTGAGACAGGGCGGCCTGGTCATGCAGAACTACAGACTGATTGACGCCACCCTGTACGTGACATTCGAGCCTTGCGTGATGTGCGCCGGCGCCATGATCCACTCTAGGATCGGCCGCGTGGTGTTTGGCGTGAGGAACTCAAAAAGAGGCGCCGCAGGCTCCCTGATGAACGTGCTGAACTACCCCGGCATGAATCACCGCGTCGAAATTACCGAGGGAATCCTGGCAGATGAATGTGCCGCCCTGCTGTGCGATTTCTATCGGATGCCTAGACAGGTGTTCAATGCTCAGAAGAAGGCCCAGAGCTCCATCAACTCCGGAGGATCTGAGGAGGCCGATACCCCAGAGAAGCTGAGGACACTGCTGGCAGAGAAGCTGTCTAGCAGGCCAGAGGCAGTGCACGAGTACGTGACCCCACTGTTCGTGTCCAGGGCACCCAATCGGAAGATGTCTGGCGCCCACAAGGACACACTGAGAAGCGCCAAGAGGTTTGTGAAGCACAACGAGAAGATCTCCGTGAAGAGAGTGTGGCTGACCGAGATCAAGCTGGCCGATCTGGAGAACATGGTGAATTACAAGAACGGCAGGGAGATCGAGCTGTATGAGGCCCTGAAGGCAAGGCTGGAGGCCTACGGAGGAAATGCCAAGCAGGCCTTCGACCCAAAGGATAACCCCTTTTATAAGAAGGGAGGACAGCTGGTGAAGGCCGTGCGGGTGGAGAAGACCCAGGAGAGCGGCGTGCTGCTGAATAAGAAGAACGCCTACACAATCGCCGACAATGGCGATATGGTGAGAGTGGACGTGTTCTGTAAGGTGGATAAGAAGGGCAAGAATCAGTACTTTATCGTGCCTATCTATGCCTGGCAGGTGGCCGAGAACATCCTGCCAGACATCGATTGCAAGGGCTACAGAATCGACGATAGCTATACATTCTGTTTTTCCCTGCACAAGTATGACCTGATCGCCTTCCAGAAGGATGAGAAGTCCAAGGTGGAGTTTGCCTACTATATCAATTGCGACTCCTCTAACGGCAGGTTCTACCTGGCCTGGCACGATAAGGGCAGCAAGGAGCAGCAGTTTCGCATCTCCACCCAGAATCTGGTGCTGATCCAGAAGTATCAGGTGAACGAGCTGGGCAAGGAGATCAGGCCATGTCGGCTGAAGAAGCGCCCACCCGTGCGGGAATTCGAGCCCAAGAAGAAGAGGAAAGTCTAA

Nme2ABE8e-797^Smu^: NLS, Cas9^Smu^, linker, TadA8e.

CCAAAGAAGAAGCGGAAAGTCGCCGCCTTCAAGCCTAACCCAATCAATTACATCCTGGGACTGGCCATCGGAATCGCATCCGTGGGATGGGCTATGGTGGAGATCGACGAGGAGGAGAATCCTATCCGGCTGATCGATCTGGGCGTGAGAGTGTTTGAGAGGGCCGAGGTGCCAAAGACCGGCGATTCTCTGGCTATGGCCCGGAGACTGGCACGGAGCGTGAGGCGCCTGACACGGAGAAGGGCACACAGGCTGCTGAGGGCACGCCGGCTGCTGAAGAGAGAGGGCGTGCTGCAGGCAGCAGACTTCGATGAGAATGGCCTGATCAAGAGCCTGCCAAACACCCCCTGGCAGCTGAGAGCAGCCGCCCTGGACAGGAAGCTGACACCACTGGAGTGGTCTGCCGTGCTGCTGCACCTGATCAAGCACCGCGGCTACCTGAGCCAGCGGAAGAACGAGGGAGAGACAGCAGACAAGGAGCTGGGCGCCCTGCTGAAGGGAGTGGCCAACAATGCCCACGCCCTGCAGACCGGCGATTTCAGGACACCTGCCGAGCTGGCCCTGAATAAGTTTGAGAAGGAGTCCGGCCACATCAGAAACCAGAGGGGCGACTATAGCCACACCTTCTCCCGCAAGGATCTGCAGGCCGAGCTGATCCTGCTGTTCGAGAAGCAGAAGGAGTTTGGCAATCCACACGTGAGCGGAGGCCTGAAGGAGGGAATCGAGACCCTGCTGATGACACAGAGGCCTGCCCTGTCCGGCGACGCAGTGCAGAAGATGCTGGGACACTGCACCTTCGAGCCTGCAGAGCCAAAGGCCGCCAAGAACACCTACACAGCCGAGCGGTTTATCTGGCTGACAAAGCTGAACAATCTGAGAATCCTGGAGCAGGGATCCGAGAGGCCACTGACCGACACAGAGAGGGCCACCCTGATGGATGAGCCTTACCGGAAGTCTAAGCTGACATATGCCCAGGCCAGAAAGCTGCTGGGCCTGGAGGACACCGCCTTCTTTAAGGGCCTGAGATACGGCAAGGATAATGCCGAGGCCTCCACACTGATGGAGATGAAGGCCTATCACGCCATCTCTCGCGCCCTGGAGAAGGAGGGCCTGAAGGACAAGAAGTCCCCCCTGAACCTGAGCTCCGAGCTGCAGGATGAGATCGGCACCGCCTTCTCTCTGTTTAAGACCGACGAGGATATCACAGGCCGCCTGAAGGACAGGGTGCAGCCTGAGATCCTGGAGGCCCTGCTGAAGCACATCTCTTTCGATAAGTTTGTGCAGATCAGCCTGAAGGCCCTGAGAAGGATCGTGCCACTGATGGAGCAGGGCAAGCGGTACGACGAGGCCTGCGCCGAGATCTACGGCGATCACTATGGCAAGAAGAACACAGAGGAGAAGATCTATCTGCCCCCTATCCCTGCCGACGAGATCAGAAATCCTGTGGTGCTGAGGGCCCTGTCCCAGGCAAGAAAAGTGATCAACGGAGTGGTGCGCCGGTACGGATCTCCAGCCCGGATCCACATCGAGACCGCCAGAGAAGTGGGCAAGAGCTTCAAGGACCGGAAGGAGATCGAGAAGAGACAGGAGGAGAATCGCAAGGATCGGGAGAAGGCCGCCGCCAAGTTTAGGGAGTACTTCCCTAACTTTGTGGGCGAGCCAAAGTCTAAGGACATCCTGAAGCTGCGCCTGTACGAGCAGCAGCACGGCAAGTGTCTGTATAGCGGCAAGGAGATCAATCTGGTGCGGCTGAACGAGAAGGGCTATGTGGAGATCGATCACGCCCTGCCTTTCTCCAGAACCTGGGACGATTCTTTTAACAATAAGGTGCTGGTGCTGGGCAGCGAGAACCAGAATAAGGGCAATCAGACACCATACGAGTATTTCAATGGCAAGGACAACTCCAGGGAGTGGCAGGAGTTCAAGGCCCGCGTGGAGACCTCTAGATTTCCCAGGAGCAAGAAGCAGCGGATCCTGCTGCAGAAGTTCGACGAGGATGGCTTTAAGGAGTGCAACCTGAATGACACCAGATACGTGAACCGGTTCCTGTGCCAGTTTGTGGCCGATCACATCCTGCTGACCGGCAAGGGCAAGAGAAGGGTGTTCGCCTCTAATGGCCAGATCACAAACCTGCTGAGGGGATTTTGGGGACTGAGGAAGGTGCGGGCAGAGAATGACAGACACCACGCACTGGATGCAGTGGTGGTGGCATGCAGCACCGTGGCAATGCAGCAGAAGATCACAAGATTCGTGAGGTATAAGGAGATGAACGCCTTTGACGGCAAGACCATCGATAAGGAGACAGGCAAGGTGCTGCACCAGAAGACCCACTTCCCCCAGCCTTGGGAGTTCTTTGCCCAGGAAGTGATGATCCGGGTGTTCGGCAAGCCAGACGGCAAGCCTGAGTTTAGCGGGGGGTCATCTGAGGTGGAGTTTTCCCACGAGTACTGGATGAGACATGCCCTGACCCTGGCCAAGAGGGCACGGGATGAGAGGGAGGTGCCTGTGGGAGCCGTGCTGGTGCTGAACAATAGAGTGATCGGCGAGGGCTGGAACAGAGCCATCGGCCTGCACGACCCAACAGCCCATGCCGAAATTATGGCCCTGAGACAGGGCGGCCTGGTCATGCAGAACTACAGACTGATTGACGCCACCCTGTACGTGACATTCGAGCCTTGCGTGATGTGCGCCGGCGCCATGATCCACTCTAGGATCGGCCGCGTGGTGTTTGGCGTGAGGAACTCAAAAAGAGGCGCCGCAGGCTCCCTGATGAACGTGCTGAACTACCCCGGCATGAATCACCGCGTCGAAATTACCGAGGGAATCCTGGCAGATGAATGTGCCGCCCTGCTGTGCGATTTCTATCGGATGCCTAGACAGGTGTTCAATGCTCAGAAGAAGGCCCAGAGCTCCATCAACTCCGGAGGATCTTTTGAGGAGGCCGATACCCCAGAGAAGCTGAGGACACTGCTGGCAGAGAAGCTGTCTAGCAGGCCAGAGGCAGTGCACGAGTACGTGACCCCACTGTTCGTGTCCAGGGCACCCAATCGGAAGATGTCTGGCGCCCACAAGGACACACTGAGAAGCGCCAAGAGGTTTGTGAAGCACAACGAGAAGATCTCCGTGAAGAGAGTGTGGCTGACCGAGATCAAGCTGGCCGATCTGGAGAACATGGTGAATTACAAGAACGGCAGGGAGATCGAGCTGTATGAGGCCCTGAAGGCAAGGCTGGAGGCCTACGGAGGAAATGCCAAGCAGGCCTTCGACCCAAAGGATAACCCCTTTTATAAGAAGGGAGGACAGCTGGTGAAGGCCGTGCGGGTGGAGAAGACCCAGGAGAGCGGCGTGCTGCTGAATAAGAAGAACGCCTACACAATCGCCGACAATGCAACCATGGTGAGAGTGGATGTGTACACAAAAGCCGGCAAAAATTATCTGGTGCCTGTGTACGTGTGGCAGGTGGCCCAGGGCATCCTGCCTAACAGAGCCGTGACAAGCGGCAAGAGCGAAGCTGATTGGGATCTGATCGATGAGTCTTTTGAGTTTAAGTTTAGCCTGTCTAGGGGCGATCTGGTGGAGATGATCAGCAATAAGGGCAGAATTTTTGGCTATTATAATGGACTGGATAGAGCTAATGGCAGCATCGGCATCAGAGAGCACGATCTGGAGAAGTCAAAGGGAAAAGATGGCGTGCACAGAGTGGGCGTGAAAACAGCCACCGCCTTCAATAAGTACCATGTGGATCCTCTGGGCAAGGAGATCCACAGGTGTAGCTCTGAGCCTAGACCAACACTGAAGATTAAGAGCAAGAAGGGCACCGGCGGGCAAAGAAGAAGCGGAAAGTCTAG

Nme2ABE8e-797^-C^: NLS, Cas9^-C^, linker, TadA8e.

CCAAAGAAGAAGCGGAAAGTCGCCGCCTTCAAGTCAAACCCAATCAATTACATCCTGGGACTGGCCATCGGAATCGCATCCGTGGGATGGGCTATGGTGGAGATCGACGAGGAGGGGAATCCTATCCGGCTGATCGATCTGGGCGTGAGAGTGTTTGAGAGGGCCGAGGTGCCAAAGACCGGCGATTCTCTGGCTATGGCCCGGAGACTGGCACGGAGCGTGAGGCGCCTGACACGGAGAAGGGCACACAGGCTGCTGAGGGCACGCCGGCTGCTGAAGAGAGAGGGCGTGCTGCAGGCAGCAGACTTCGATGAGAATGGCCTGATCACGAGCCTGCCAAACACCCCCTGGCAGCTGAGAGCAGCCGCCCTGGACAGGAAGCTGACACCACTGGAGTGGTCTGCCGTGCTGCTGCACCTGATCAAGCACCGCGGCTACCTGAGCCAGCGGAAGAACGAGGGAGAGACAGCAGCCAAGGAGCTGGGCGCCCTGCTGAAGGGAGTGGCCAACAATGCCCACGCCCTGCAGACCGGCGATTTCAGGACACCTGCCGAGCTGGCCCTGAATAAGTTTGAGAAGGAGTCCGGCCACATCAGAAACCAGAGGGGCGACTATAGCCACACCTTCTCCCGCAAGGATCTGCAGGCCGAGCTGATCCTGCTGTTCGAGAAGCAGAAGGAGTTTGGCAATCCACACGTGAGCGGAGGCCTGAAGGAGGGAATCGAGACCCTGCTGATGACACAGAGGCCTGCCCTGTCCGGCGACGCAGTGCAGAAGATGCTGGGACACTGCACCCTCGAGCCTACAGAGCCAAAGGCCGCCAAGAACACCTACACAGCCGAGCGGTTTATCTGGCTGACAAAGCTGAACAATCTGAGAATCCTGGAGCAGGGATCCGAGAGGCCACTGACCGACACAGAGAGGTCCACCCTGATGGATGAGCCTTACCGGAAGTCTAAGCTGACATATGCCCAGGCCAGAAAGCTGCTGGGCCTGGAGGACACCGCCTTCTTTAAGGGCCTGAGATACGGCAAGGATAATGCCGAGGCCTCCACACTGATGGAGATGAAGGCCTATCACGCCATCTCTCGCGCCCTGGAGAAGGAGGGCCTGAAGGACAAGAAGTCCCCCCTGAACCTGAGCTCCGAGCTGCAGGATGAGATCGGCACCGCCTTCTCTCTGTTTAAGACCGACGAGGATATCACAGGCCGCCTGAAGGACAGGGTGCAGCCTGAGATCCTGGAGGCCCTGCTGAAGCACATCTCTTTCGATAAGTTTGTGCAGATCAGCCTGAAGGCCCTGAGAAGGATCGTGCCACTGATGGAGCAGGGCAAGCGGTACGACGAGGCCTGCGCCGAGATCTACGGCGTTCACTATGGCAAGAAGAACACAGAGGAGAAGATCTATCTGCCCCCTATCCCTGCCGACGAGATCAGAAATCCTGTGGTGCTGAGGGCCCTGTCCCAGGCAAGAAAAGTGATCAACGGAGTGGTGCGCCGGTACGGATCTCCAGCCCGGATCCACATCGAGACCGCCAGAGAAGTGGGCAAGAGCTTCAAGGACCGGAAGGAGATCGCGAAGAGACAGGAGGAGAATCGCAAGGATCGGGAGAAGGCCGCCGCCAAGTTTAGGGAGTACTTCCCTAACTTTGTGGGCGAGCCAAAGTCTAAGGACATCCTGAAGCTGCGCCTGTACGAGCAGCAGCACGGCAAGTGTCTGTATAGCGGCAAGGAGATCAATCTGGTGCGGCTGAACGAGAAGGGCTATGTGGAGATCGATCACGCCCTGCCTTTCTCCAGAACCTGGGACGATTCTTTTAACAATAAGGTGCTGGTGCTGGGCAGCGAGAACCAGAATAAGGGCAATCAGACACCATACGAGTATTTCAATGGCAAGGACAACTCCAGGGAGTGGCAGGAGTTCAAGGCCCGCGTGGAGACCTCTAGATTTCCCAGTAGCAAGAAGCAGCGGATCCTGCTGCAGAAGTTCGACGAGGATGGCTTTAAGGAGTGCAACCTGAATGACACCAGATACGTGAACCGGTTCCTGTGCCAGTTTGTGGCCGATCACATCCTGCTGACCGGCAAGGGCAAGAGAAGGGTGGTCGCCTCTAATGGCCAGATCACAAACCTGCTGAGGGGATTTTGGAGACTGAGGAAGGTGCGGGCAGAGAATGACAGACACCACGCACTGGATGCAGTGGTGGTGGCATGCAGCACCGTGGCAATGCAGCAGAAGATCACAAGATTCGTGAGGTATAAGGAGATGAACGCCTTTGACGGCAAGACCGTCGATAAGGAGACAGGCAAGGTGCTGTACCAGAAGACCCACTTCCCCCAGCCTTGGGAGTTCTTTGCCCAGGAAGTGATGATCCGGGTGTTCGGCAAGCCAGACGGCAAGCCTGAGTTTAGCGGGGGGTCATCTGAGGTGGAGTTTTCCCACGAGTACTGGATGAGACATGCCCTGACCCTGGCCAAGAGGGCACGGGATGAGAGGGAGGTGCCTGTGGGAGCCGTGCTGGTGCTGAACAATAGAGTGATCGGCGAGGGCTGGAACAGAGCCATCGGCCTGCACGACCCAACAGCCCATGCCGAAATTATGGCCCTGAGACAGGGCGGCCTGGTCATGCAGAACTACAGACTGATTGACGCCACCCTGTACGTGACATTCGAGCCTTGCGTGATGTGCGCCGGCGCCATGATCCACTCTAGGATCGGCCGCGTGGTGTTTGGCGTGAGGAACTCAAAAAGAGGCGCCGCAGGCTCCCTGATGAACGTGCTGAACTACCCCGGCATGAATCACCGCGTCGAAATTACCGAGGGAATCCTGGCAGATGAATGTGCCGCCCTGCTGTGCGATTTCTATCGGATGCCTAGACAGGTGTTCAATGCTCAGAAGAAGGCCCAGAGCTCCATCAACTCCGGAGGATCTGAGGAGGCCGATACCCCAGAGAAGCTGAGGACACTGCTGGCAGAGAAGCTGTCTAGCAGGCCAGAGGCAGTGCACGAGTACGTGACCCCACTGTTCGTGTCCAGGGCACCCAATCGGAAGATGTCTGGCGCCCACAAGGACACACTGAGAAGCGCCAAGAGGTTTGTGAAGCACAACGAGAAGATCTCCGTGAAGAGAGTGTGGCTGACCGAGATCAAGCTGGCCGATCTGGAGAACATGGTGAATTACAAGAACGGCAGGGAGATCGAGCTGTATGAGGCCCTGAAGGCAAGGCTGGAGGCCTACGGAGGAAATGCCAAGCAGGCCTTCGACCCAAAGGATAACCCCTTTTATAAGAAGGGAGGACAGCTGGTGAAGGCCGTGCGGGTGGAGAAGACCCAGAAGAGCGGCGTGCTGCTGAATAAGAAGAACGCCTACACAATCGCCGACAATGGCGATATGGTGAGAGTGGACGTGTTCTGTAAGGTGGATAAGAAGGGCAAGAATCAGTACTTTATCGTGCCTATCTATGCCTGGCAGGTGGCCGAGAACATCCTGCCAGACATCGATTGCAAGGGCTACAGAATCGACGATAGCTATACATTCTGTTTTTCCCTGCACAAGTATGACCTGATCGCCTTCCAGAAGGATGAGAAGTCCAAGGTGGAGTTTGCCTACTATATCAATTGCGACTCCTCTAGCGGCGGGTTCTACCTGGCCTGGCACGATAAGGGCAGCAGGGAGCAGCGGTTTCGCATCTCCACCCAGAATCTGGCGCTGATCCAGAAGTATCAGGTGAACGAGCTGGGCAAGGAGATCAGGCCATGTCGGCTGAAGAAGCGCCCACCCGTGCGGGAATTCGAGCCCAAGAAGAAGAGGAAAGTCTAA
