# Supplementary figures and images for: Engineered domain-inlaid Nme2Cas9 adenine base editors with increased on-target DNA editing and targeting scope
Source: BMC Biol. 2023 Nov 9;21:250. doi: 10.1186/s12915-023-01754-4 (PMC10636962; doi:10.1186/s12915-023-01754-4)

**Raw Images for Figure 3d.**


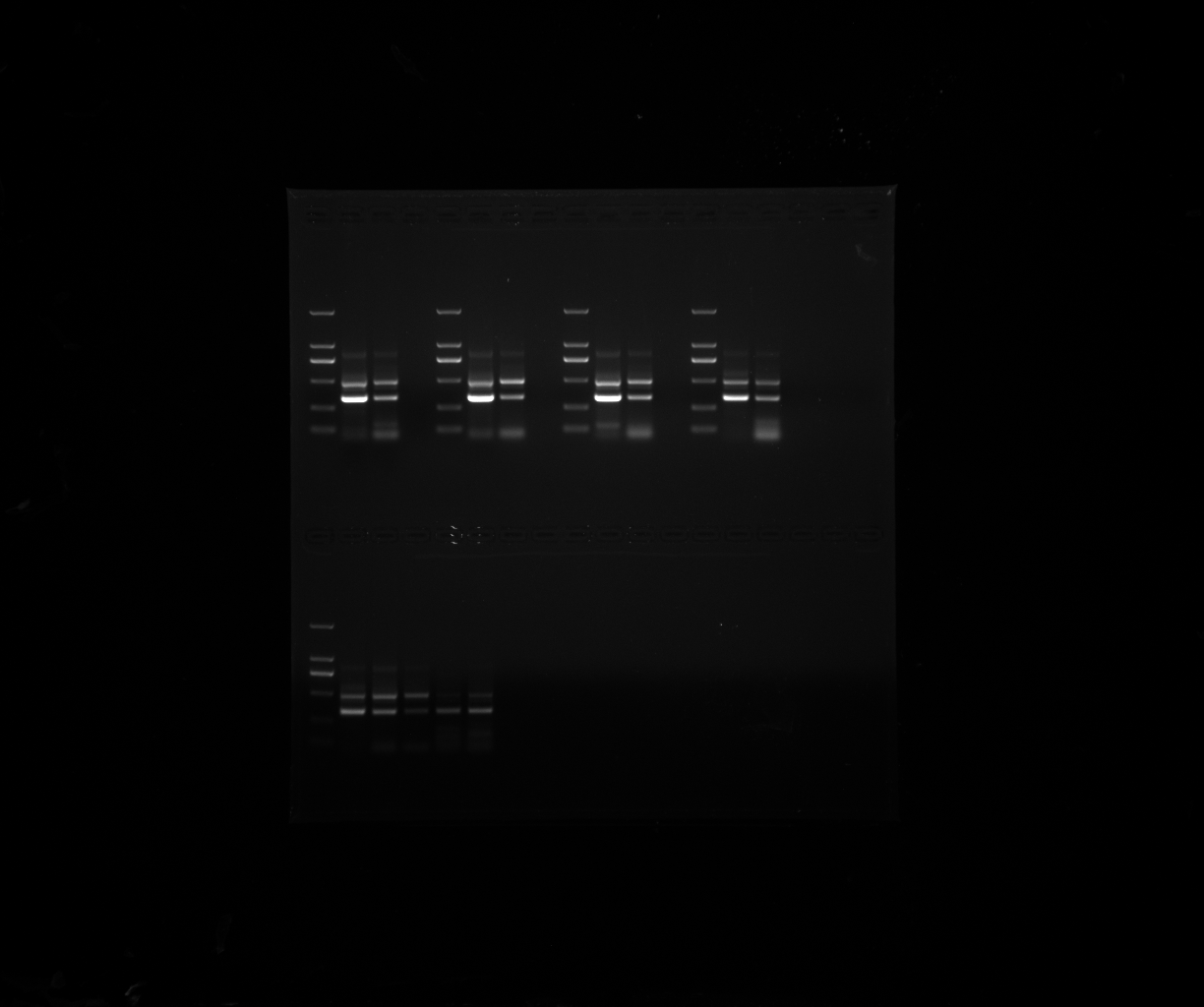

Supplement: Supplementary file 3 — Additional file 3. Raw Images for Fig. 3d. [file 12915_2023_1754_MOESM3_ESM.docx]
